# Identification and Validation of Pathogenic Genes in Sepsis and Associated Diseases by Integrated Bioinformatics Approach

**DOI:** 10.3390/genes13020209

**Published:** 2022-01-24

**Authors:** Mohd Murshad Ahmed, Almaz Zaki, Alaa Alhazmi, Khalaf F. Alsharif, Hala Abubaker Bagabir, Shafiul Haque, Kailash Manda, Shaniya Ahmad, Syed Mansoor Ali, Romana Ishrat

**Affiliations:** 1 Centre for Interdisciplinary Research in Basic Sciences, Jamia Millia Islamia, New Delhi 110025, India; murshad.60ali@gmail.com; 2 Translational Research Lab, Department of Biotechnology, Faculty of Natural Sciences, Jamia Millia Islamia, New Delhi 110025, India; almazzaki1122@gmail.com (A.Z.); ahmadshaniya@gmail.com (S.A.); 3 Medical Laboratory Technology Department, SMIRES for Consultation in Specialized, Jazan University, Jazan 45142, Saudi Arabia; alaa.alhazmi@gmail.com; 4 Department of Clinical Laboratory Sciences, College of Applied Medical Sciences, Taif University, Taif 21944, Saudi Arabia; alsharif@tu.edu.sa; 5 Department of Medical Physiology, Faculty of Medicine, King Abdulaziz University, Rabigh 21589, Saudi Arabia; habajaber@kau.edu.sa; 6 Research and Scientific Studies Unit, College of Nursing and Allied Health Sciences, Jazan University, Jazan 45142, Saudi Arabia; shafiul.haque@hotmail.com; 7 Institute of Nuclear Medicine and Applied Sciences, Defense Research Development Organization, New Delhi 110054, India; kailashmandal@gmail.com

**Keywords:** sepsis, cardiorenal syndrome, systemic lupus erythematosus, differentially expressed genes, miRNA

## Abstract

Sepsis is a clinical syndrome with high mortality and morbidity rates. In sepsis, the abrupt release of cytokines by the innate immune system may cause multiorgan failure, leading to septic shock and associated complications. In the presence of a number of systemic disorders, such as sepsis, infections, diabetes, and systemic lupus erythematosus (SLE), cardiorenal syndrome (CRS) type 5 is defined by concomitant cardiac and renal dysfunctions Thus, our study suggests that certain mRNAs and unexplored pathways may pave a way to unravel critical therapeutic targets in three debilitating and interrelated illnesses, namely, sepsis, SLE, and CRS. Sepsis, SLE, and CRS are closely interrelated complex diseases likely sharing an overlapping pathogenesis caused by erroneous gene network activities. We sought to identify the shared gene networks and the key genes for sepsis, SLE, and CRS by completing an integrative analysis. Initially, 868 DEGs were identified in 16 GSE datasets. Based on degree centrality, 27 hub genes were revealed. The gProfiler webtool was used to perform functional annotations and enriched molecular pathway analyses. Finally, core hub genes (*EGR1, MMP9*, and *CD44*) were validated using RT-PCR analysis. Our comprehensive multiplex network approach to hub gene discovery is effective, as evidenced by the findings. This work provides a novel research path for a new research direction in multi-omics biological data analysis.

## 1. Introduction

Sepsis is a cause of high mortality and morbidity even in the most developed and wealthiest countries. Globally, 31.5 million patients with sepsis and 19.4 million instances of severe sepsis are expected to be diagnosed each year, with 5.3 million deaths possible [1]. In sepsis, the abrupt release of cytokines by the innate immune system may cause multiorgan failure, leading to septic shock and associated complications, including CRS [2,3,4] and an autoimmune disease known as SLE [5,6,7]. Cardiovascular injury and acute kidney injury (AKI) have been shown to be independent risk factors for increased mortality [8]. Combined cardiac and renal dysfunction amplifies the progression of failure in the individual organ, ultimately leading to the failure of both organs (heart and kidney), often referred as CRS, while SLE is a systemic autoimmune disease that occurs primarily due to poor efferocytosis, uncontrolled complement activation, complex immune redundancy, and systemic inflammation [9]. To avoid the unnecessary and tedious usage of antimicrobial drugs in sepsis, early and distinctive detection is required. Conventional biomarkers for sepsis, SLE, and CRS are not accurate and adequate due to the chance of false positive results and delayed detection [10,11,12]. Therefore, more reliable biomarkers for early detection and appropriate therapeutic targets are needed to decrease risk assessment. Elevated inflammatory cytokines, such as *TNF-α*, *IL-1β*, and *IL-6*, during sepsis do not provide specific information regarding the development and severity of the disease, and hence considering them as specific disease clinical markers is not suitable [13]. It has been reported that patients with sepsis and SLE can develop type 5 CRS, where all of these disorders can lead to disease in the heart and kidneys [14]. The severity of the illness, the occurrence of septic shock, and mortality are greater in patients with septic CRS-5 compared with patients who only have sepsis [2]. All vital organs of the body share biological information, which is termed organ cross-talk. CRS is an important example of organ cross-talk, whereby interdependency exists between the function of the kidney and heart and vice versa [9,15,16] Mostly, CRS is associated with cardiovascular disease (CVD) and chronic kidney disease (CKD), both of which are intimately linked. Early detection and effective treatments are the only ways to lower the death rates caused by chronic diseases [17]. Due to the slow progression of chronic diseases (CDs), it is critical to make an early diagnosis and administer efficient treatment. As a result, it is critical to develop a decision model that may aid in the diagnosis of CDs and the prediction of future patient outcomes [18]. We choose a broad term using the plural CRS to indicate the presence of multiple syndromes. For instance, in sepsis-induced acute CRS-5, there is a fulminant disease process that has a dramatic impact on both the kidneys and heart [19]. The microarray data profiling of sepsis, CRS, and SLE, and extensive gene techniques, have been used to identify differentially expressed genes (DEGs) [20,21,22,23]. Incorporating, integrating, and re-analyzing existing data using bioinformatics tools can provide new strategies, methods, and relevant information. In network biology, there are numerous approaches to this [18].

Despite tremendous advances in the diagnosis, treatment, and prognosis of CVD, new diagnostic biomarkers and therapeutic techniques are still needed to reduce the prevalence of these diseases [24], especially the ones that cover metabolic syndrome, which is a group of conditions that includes diabetes and obesity. Biomarkers will be crucial in the development of customized therapeutics for CVD [25]. Circulating microRNAs (miRNAs) have been evaluated as emerging biomarkers for CVD, CRS, or renal diseases, and can be used as a new therapeutic target [26,27,28]. 

The determination of essential and significant proteins in the network plays an indispensable role in understanding and developing therapeutic strategies in a particular disease. In this study, our current paradigm for studying CRS revolves around identifying the key regulatory genes shared by sepsis and SLE and CRS and SLE, from a selection of manually curated genes, by combining protein regulatory interactions, functions, and disease networks, identifying those that can be employed as critical genes in future research. The study is also confined to the topological properties of regulatory networks, their activities, and mechanisms, which will ultimately enable us to predict key regulatory miRNAs and genes among the millions of genes that could be targeted in the therapeutic treatment of these diseases.

## 2. Research Design (Methodology)

### 2.1. Acquisition and Pre-Processing of Data

**Figure 1:**
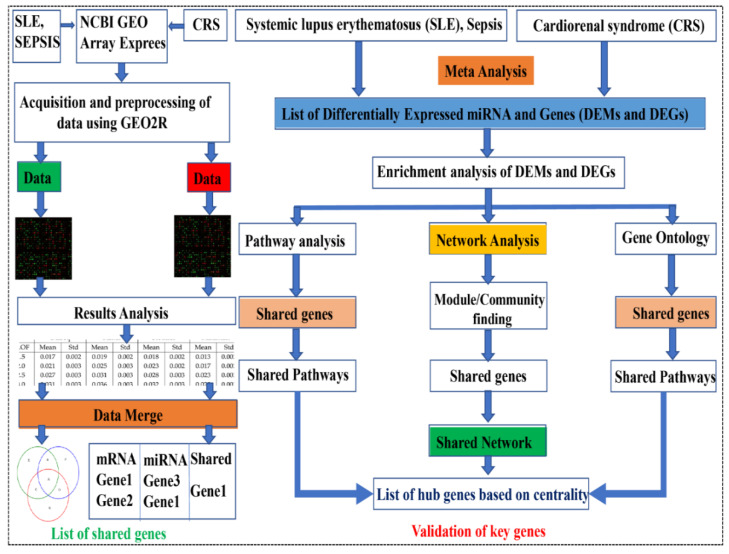
Methodology adopted for the series used in the study. SLE, sepsis, and CRS data were extracted from NCBI geo datasets, and these terms were also searched for in the Array Express database. The GSE series were available in the public databases, and we retrieved only human data by applying exclusion criteria. Data were filtered via GEO2R and selected after final mining, applying normalization and log 2 transformations. The resulting data are in the Excel sheet used for DEGs (based on fold change and *p*-value). The data were merged to find overlapping DEGs and DEMs, and finally, the PPIN network was constructed via overlapping DEGs-DEMs (shared network).

The mRNA expression profiles of SLE, CRS, and sepsis, as well as the CRS miRNA expression profile, were obtained from the NCBI’s Gene Expression Omnibus (GEO) (www.ncbi.nlm.nih.gov/geo/, accessed on 10 May 2021). Six SLE GSE series (GSE30153, GSE50772, GSE51997, GSE13887, GSE99967, GSE103760), six sepsis GSE series (GSE6535, GSE5772, GSE28750, GSE64457, GSE12624, GSE13205), and four CRS GSE series were extracted (GSE17582 and GSE125898 contain mRNA data, while GSE series GSE89699 and GSE87885 contain data relating to miRNAs). The details of all GSE series are provided in Table 1. The GSE series data were collected and pre-processed considering the standard protocol, as described in Figure 1. The keywords searched in the GEO datasets were “SLE (systemic lupus erythematous), Sepsis, septic shock, neonatal sepsis, CRS (Cardiorenal Syndrome), syndrome, and heart and kidney disease”, with human sources and excluding SRA-run data (NGS Data). Some GSE series data have other diseases in the sample, such as MBD and diabetes. Therefore, we chose only the GSE series that had the disease of interest (sepsis, SLE, and CRS), as shown in Tables S1 and S2. The inclusion and exclusion criteria were applied to each of the 16 datasets. The current study contained a total of 224/409 (healthy/patients) samples. The Illumina HumanRef-8 v3.0 expression bead chip, Agilent-046064 Unrestricted_Human_miRNA_V19.0_Microarray, Affymetrix Human Genome U133 Plus 2.0 Array, Affymetrix Human Genome U133A Array, NHICU Human 19K v1.0, Affymetrix 2.0 microarray, LC Sciences μParaflo human miRNA array, Affymetrix Human Gene 2.0 ST microarray platforms were used in the selected datasets. The microarray research also included PBMC, monocyte, B cells, T cells, among other tissues. Patient samples from various sources were not differentiated during the data integration procedure in order to show a common/overlapped gene signature. Table 1 lists the specific details for each dataset, including the GEO accession number, the total number of samples (normal and disease samples) illness, platform, and authors. Gene expression patterns differ in different organs and tissues of the body, and complex metabolic disorders, including diabetes, obesity, and sepsis, etc., are influenced by gene expression patterns across a wide range of organs and tissues. The expression microarray is a method for studying gene expression on a genome-wide scale that is widely utilized. Nevertheless, the studies that are published on a yearly basis by thousands of scientists performing microarray experiments are tainted by the systemic inaccuracy known as batch effects. Batch effects can be decreased through proper experimental design, but they cannot be eradicated until the entire study is conducted in one batch [29]. Before analyzing the microarray data, a number of algorithms were available to correct batch effects. We employed the empirical Bayes method built-in function in limma, in combination with the fit2 function. Meta-analyses based on microarray data integration require effective in silico methods. We may now use in silico tools to efficiently merge numerous microarray datasets while ignoring differing demographics, experimental designs, and specimen sources, with the advancements of ever-growing theories and bioinformatics tools [30].

### 2.2. Normalization of Raw Data

The majority of the data submitted to the NCBI or other databases, such as Array Express and Affymetrix, are unnormalized. Data may not be normalized due to data noise, redundancy, duplication, and the values of outliers. Data that have been normalized for the accuracy and dependability of results reduces these errors. If we proceed with data without normalization, the results of the DEGs may differ (false results). In R, there are several methods for normalizing data (MAS5, RMA, and GCRMA) [15]. For background correction and data normalization, we used the RMA (robust multi-array) method (see the R script below). The boxplots of the GSE series before and after normalization show the differences in expression. The data log 2 transforms were used in the normalization process to remove outliers—the Benjamin Hackenberg methods are used by default for p-value correction. We used the limma package in the GEO2R and R platforms to find DEGs. For DEGs selection, various old methods (Edges, Seq2. Rank methods) were available in the R packages. However, limma is the most reliable and usable method for microarray data analysis [41]. Because different probe ids have the same gene name, the results may be perplexing. Furthermore, many genes have alternate names (synonyms). In the STRING app (version 11.3), there were no (false genes) data available. There was a significant probe level effect after applying RMA to the data and noting that the probe set expression values among the replicates correlated no better than among non-replicates. We reduced the amount of total variation while maintaining diversity between couples by standardizing means and variances across replicate groups. After normalization, there was no association between non-replicates. As a result, all of these issues were filtered during the data processing and prior to the final hub gene discovery. The probe numbers from the expression profile were converted into gene symbols. The robust multi-array average in the R affy package was used to perform background correction and quartile data normalization (Affymetrix, Sunnyvale, CA, USA). The convolution background was corrected, the missing values were estimated, and the expression values were log2 transformed and normalized during pre-processing. Genes with a *p*-value < 0.05 and a fold change greater than |0.5–2| were chosen as differentially expressed genes (DEGs) between control and treatment samples.

>library(Biobase)

>library(limma)

>library(affy)

> data = ReadAffy()

>boxplot(data,which = ′pm′, col = ′red′)

>boxplot(expr.rma, col = c(1:12))

> affy.bg = bg.correct(data, method = ″rma″)

> boxplot(affy.bg, col = rainbow(8))

>affy.norm = normalize(affy.bg, method = ″quantiles″)

>boxplot(affy.norm, col = rainbow(8))

### 2.3. Meta-analysis of Differentially Expressed Genes (DEGs)

The GSE17582, GSE99967, and GSE103760 obtained data samples series were already normalized. The other 13 GSE series for the three conditions (sepsis, SLE, and CRS) were processed for further analysis through the GEO2R (http://www.ncbi.nlm.nih.gov/geo/geo2r/, accessed on 5 June 2021) tool. This is a web-based analytical tool with an in-built linear models for microarray data (limma) R package and GEO query. Default parameters were applied for the pre-processing of datasets. Differentially expressed microRNAs were extracted using criteria *p* < 0.05 and |log fold-change| > 0.5 to 2 as the threshold values. The up- and downregulated genes in each GSE series were overlapped in order to find meta-analysis-critical genes that were used as shared genes in the PPIN network. The upregulated genes from all sepsis series were submitted to Bioinfogps in order to find overlapping genes (an online tool for finding common values and displaying Venn diagrams). The same procedure was used for all the downregulated genes in the sepsis, CRS, and SLE series. The final list of overlapping DEGs was used to build a network for up- and downregulated DEGs separately.

### 2.4. Prediction of Target Genes of Differentially Expressed miRNAs (DEMs)

Four different approaches have been developed for the in silico target prediction of DEMs. In the past five years, around twenty-five miRNA target prediction algorithms for mammalian genomes have been reported so far. Of all of the programs that have been developed, we used: TargetScan (http://www.targetscan.org/vert_72/, accessed on 15 June 2021). This predicts miRNA targets from multiple genomes. These algorithms compare multiple genomes to predict targets [42]. miRmap (https://mirmap.ezlab.org/, accessed on 16 June 2021) is an open-source, freely available python library that uses web facilities to predict miRNA targets [43]. miRWalk (version 3.0) (http://mirwalk.umm.uni-heidelberg.de/, accessed on 17 June 2021) is based on a computational approach, and is coded in the Perl programming language to predict target sites [44].

Moreover, the mirDIP (http://ophid.utoronto.ca/mirDIP/, accessed on 17 June 2021) database provides comprehensive, reliable, and user-friendly resources to predict miRNA targets. Many users use mirDIP even with less knowledge of statistical analysis or computational approaches [45]. Predicted genes shared across the four databases were considered to be target genes.

### 2.5. Protein–Protein Interaction (PPI) and DEmiRNA–Gene Network Construction

The DEGs were submitted to the STRING database with a confidence score of <0.9. Cytoscape 3.8.1 or above was used to import the network. The probe IDs of the genes were mapped to their corresponding gene symbols, and their related functional information was collected to build the core network of DEGs. The gene regulatory network for SLE, sepsis, and CRS was built using the simple notion of one gene, one protein. There are a number of features that can be utilized to build and filter a network. Because the networks can be formed around a specific gene or illness, they combine data from curated databases with information from the literature. The Cytoscape plugin, molecular complex detection (MCODE; version 1.31), and the Cytoscape plugin, cytoHubba (version 0.1), were adopted to identify the significant modules’ top-ranked genes in the PPI network. The network’s topological properties were calculated using Network Analyzer, a plug-in in Cytoscape version 3.8.2, while for eigenvalue calculation, we used CytoNCA, another plug-in in Cytoscape for topological properties calculation. This could further help cross-check the results of Network Analyzer.

### 2.6. Characterization of Topological Properties of Networks

The behavior of topological parameters was utilized to characterize the structural characteristics of complex networks. The topological aspects of the network (graph) were researched to study the network’s basic behaviors: degree distribution, neighborhood connectivity, clustering coefficient, and eigenvector centrality are all described using terminology such as betweenness centrality, closeness centrality, and eigenvector centrality.

### 2.7. Module/Community Finding: MCODE Method

MCODE, the mechanism for detecting modules/communities, was used in Cytoscape. Modules from a whole network, and sub-modules from modules at each level of organization, were identified until only motifs remained (i.e., three nodes and three edges). The MCODE plugin is a popular tool for locating clusters in a network. To do so, it iteratively adds surrounding vertices with similar weights to construct clusters (strong nodes connections) from a vertex using vertex weighting (a type of clustering coefficient). “Degree cutoff = 2”, “node score cutoff = 0.2”, “*k*-score = 2”, and “max. depth = 100” were used as default values in MCODE settings for network scoring and cluster finding. These modules were also tracked in order to locate our DEG(s) of interest.

### 2.8. Gene Ontology and Pathway Analysis

Gene ontology terms give us a controlled vocabulary of terms that are divided into three categories: molecular function, biological processes, and protein class [46]. As a result, gProfiler, an online database that can augment a set of DEGs with GO keywords, was used to conduct a preliminary investigation into the functional differences among the DEGs collected. To analyze target mRNA function and define the GO term, we employed gene ontology using gprofile, an ensemble database, an enhanced database, and GOnet. The DEG(s) involved in the shared PPIN clusters were subjected to the pathway and GO word enrichment analysis. The top 10 routes and GO word hits were chosen for further examination within this range, with a statistically significant cutoff of 0.05.

### 2.9. Experimental Mice

Eight C57BL/6J wild-type mice (age: 8–10 weeks, weight: 20–25 g) were obtained from an in-house inbreeding facility at the Institute of Nuclear Medicine and Allied Sciences (INMAS) Defence Research Development Organization (DRDO), New Delhi. The institute’s animal ethics committee (IAEC) (INM/IAEC/2018/25/ext) approved the experimental study and protocols, and they conducted their procedure according to the appropriate guidelines. Mice were housed in a temperature-controlled condition room (22–25 °C temperature), on a light/dark 12 h cycle, with access to food and water ad libitum. 

### 2.10. Experimental Model Protocol

The experimental mice were allocated into two groups: sepsis (CLP-operated) and control (SHAM-operated). Briefly, mice were anesthetized first with an intraperitoneal administration of ketamine and xylazine (90 mg and 10 mg/kg, respectively). After adequate anesthesia, the lower quadrants of the mice’s abdomens were shaved, and the surgery area was disinfected. A longitudinal incision was made, and the cecum was located, exteriorized, and ligated. After ligation, the cecum was perforated by a puncture (through and through), fecal material was extruded, and then the cecum was relocated. Peritoneum and skin were sealed via a running suture (absorbable 4.0 silk and non-absorbable 4.0 silk suture). Betadine was applied all over the surgery area after operation, and saline was administered subcutaneously for fluid resuscitation. Control (SHAM-operated) mice underwent the same procedure without puncture and ligation. After sixteen hours of operation, the mice were sacrificed, and lung tissues were harvested, snap frozen, and stored at −80 °C until RNA extraction was performed.

### 2.11. Extraction of RNA and qRT-PCR

According to the manufacturer’s procedure details, RNA was extracted from the lung tissue using a Trizol reagent (Ambion, Carlsbad, CA, USA). RNA concentration was quantified using nanodrop, and 1000 ng of RNA was reverse transcribed into cDNA using Bio-Rad’s iScript cDNA synthesis kit. The reaction protocol for cDNA synthesis involved: priming at 25 °C for 5 min, reverse transcription at 46 °C for 20 min, and reverse transcriptase inactivation at 95 °C for 1 min. qRT-PCR analysis was conducted for pathogenic genes *MMP9, CD44*, and *EGR1*, which were obtained through our integrated bioinformatics approach using iTaq Universal SYBR Green Supermix (Bio-Rad, Hercules, California city, USA). The PCR analysis was conducted using the StepOne real-time PCR system. Actin was used here as an endogenous control. The Livak method (∆∆Ct) method was used for calculating the relative fold change in expression levels. Primers for respective genes were obtained from Sigma (Table 2). 

### 2.12. Statistical Analysis 

Statistical data analysis was conducted using GraphPad Prism 8 software (San Diego, CA, USA). All the results are represented as mean ± standard error of the mean (SEM). The statistical significance of the data was analyzed using Student’s *t*-test for comparing two experimental groups. Statistical significance was taken at * *p* < 0.05 and *** *p* < 0.01.

## 3. Results

### 3.1. Normalization of Data Using R

**Figure 2:**
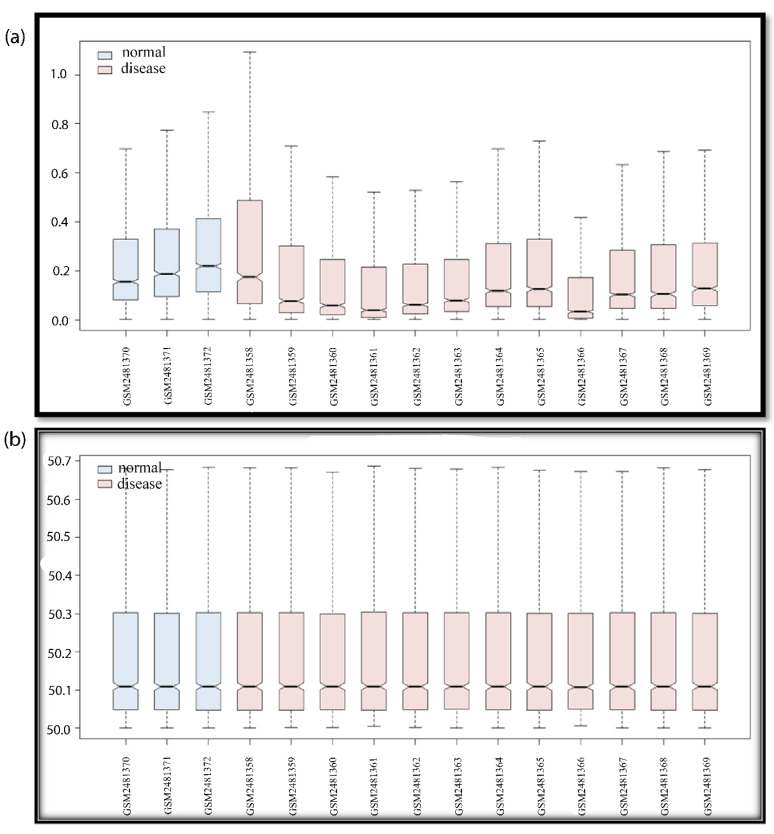
Boxplot of GSE94717. For all GSE series, the boxplot indicates data noise/data duplicity and normalized/non-normalized data. If the data are not normalized, the whisker boxes are up and down (not in proper lines). If data are random due to noise mixing or redundancy, data should be normalized in order to solve this problem. If data are non-normalized, there may be false results; the upregulated genes may be portrayed as downregulated, and vice-versa. After data are normalized, we can rely on the data, and consider the DEGs for further studies. The blue color box plot shows the normal samples and the pink color box plot shows the disease samples. In the boxplot, we can see the number of samples and the GSM number of the GSE series (if the GSE series selection was wrong, then the 12 disease samples and the 3 control samples will also give false results). The pre-processing of the data is an essential step for data accuracy when working with manipulated data. The Y-axis of the boxplot indicates a range of data values, and the X-axis indicates data samples in the GSE series. (**a**) Non-normalized data; (**b**) normalized data.

In the box plots of data, the difference in the expression values is clearly visible (see Figure 2). Before normalization, the data are randomly distributed, but after normalization, the data align. The non-normalized GSE series were downloaded from the NCBI in .cel format and saved in a folder for the RMA package in R before the data for the PPIN network were filtered using GEO2R. The resulting DEGs were evaluated and cross-checked in the GSE series literature, where genes, upregulated and downregulated, are published. If our data reveal down expression for any genes that are described as upregulated in the GSE series linked study, then this indicates that we chose the wrong samples. If the reported genes and our discovered genes both have similar expression levels, either up or down, then this verifies the study. A variety of graphs (density plot, volcano plot, and MA plot) were employed to demonstrate data accuracy, data types, and data expressions. The boxplot (whisker plot) is widely used due to its simplicity, clarity, and data accuracy. Data will not be taken for further analysis if they are non-normalized and no normalizing methods are available.

### 3.2. Gene Network Construction

**Figure 3:**
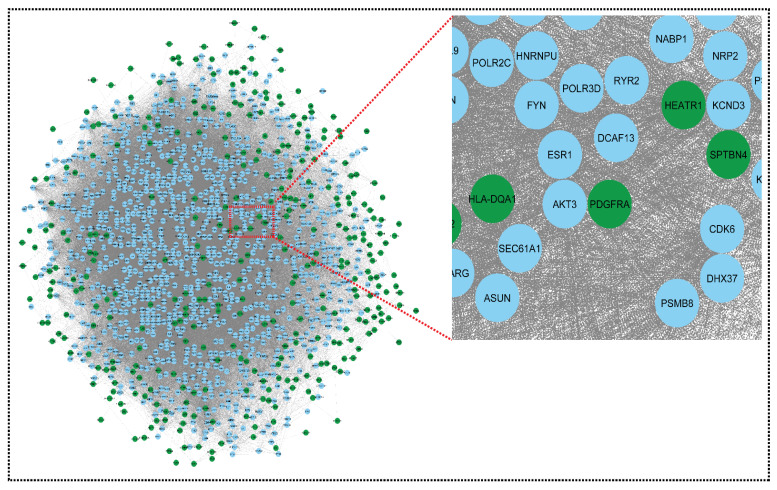
Network representing downregulated genes of sepsis, SLE, and CRS. This downregulated network contains 1351 nodes and 52,455 edges. The CRS GSE series GSE125898 contains 422 downregulated genes and GSE17582 contains 10 downregulated genes. Sepsis and SLE together contribute 13 downregulated genes. Green colors indicate our seed genes and blue are their interacting partner. A zoom view shows clear gene–gene interactions (green colors for seed genes). The nodes are the gene entity, and the edges are the interactions/connections. A gene has multiple connections in the network: gene to gene, gene to itself, and gene to many genes interactions. This complex network is undirected (no direction of edges) and unweighted. To retrieve biological information from the complex network, module analysis methods are used, which reduces complexity.

**Figure 4:**
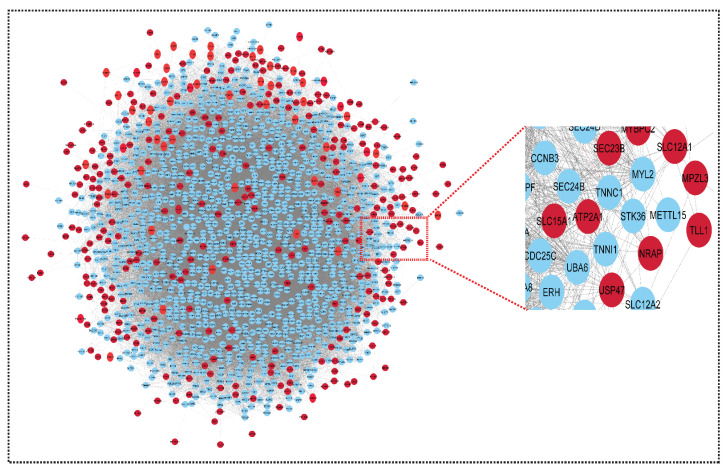
Network representing upregulated genes of sepsis, SLE and CRS. This upregulated network contains 1348 nodes and 45,699 edges. The CRS GSE series GSE125898 contains 362 upregulated genes and GSE17582 contains 11 upregulated genes. Sepsis and SLE together contribute 47 upregulated genes. The red color indicates our seed genes, and blue are their interacting partner. A highlighted (zoom view) in the right corner highlights the clear interaction between seed genes and non-seed genes. The nodes are the genes, and edges are the interactions/connections (G = V, E).

Networks are a rich source of biomarkers for disease classification because they combine mRNA profiling with protein networks to generate subnetwork biomarkers (interconnected genes whose aggregated expression levels are disease state predictive). Starting with an initial list of seed genes (47 upregulated and 13 downregulated in SLE and sepsis, although there were no overlapping genes in the two mRNA GSE series for CRS, GSE17582 (10 downregulated and 11 upregulated) and GSE125898 (10 downregulated and 11 upregulated)), we built an interaction network for CRS, SLE, and sepsis to infer gene–disease associations from network properties (with 362 upregulated and 422 downregulated genes) (see Table S3). Finally, three DEMs out of 252 were overlapped in two GSE series. They are, therefore, known to be related to the disease. In the networks, we marked the seed genes names so that each gene is represented by a single node in the interaction network, enabling us to construct a disease-specific gene interaction network, where the nodes are the seed genes and their neighbors, and the edges are the links between the nodes where there is a known interaction. The STRING database generated a network with a confidence score of 0.9, with 1348 nodes and 45,699 edges for the upregulated network and 1351 nodes and 52,455 edges for the downregulated network, shown in Figure 3 and Figure 4.

**Figure 5:**
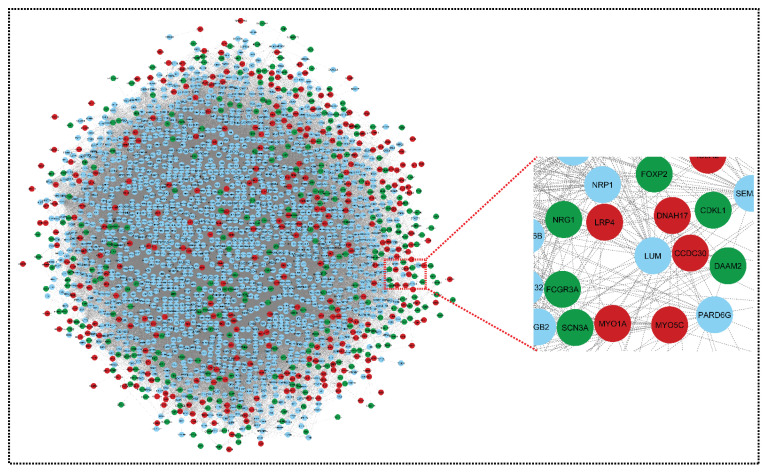
Merged network of upregulated genes and downregulated genes. This network comprises 2091 nodes and 75,957 edges. Green colors nodes and red colors nodes indicates our key genes (downregulated and upregulated, respectively), and blue nodes are their interacting partners (other genes). Nodes are the gene entity and edges are the interactions/connections. This merged network is prepared using the Network Analyzer function in Cytoscape. The genes have a common connection in both networks and merge into one network: both networks, upregulated and downregulated, have 1306 and 1348 nodes, respectively; after merging, the total genes should be 2698, but in the merged network, this becomes 2091, because some of the genes are the same in both networks (i.e., we removed duplicate genes).

Using the STRING results tab, we saved the networks as text and imported them into Cytoscape version 3.8.2. The probe IDs for the genes were mapped to their corresponding gene symbols, and their related functional information was collected in order to build the core network of differentially expressed genes. The gene regulatory network for CRS, SLE, and sepsis was built using the simple premise of one gene, one protein. Both networks could be imported into Cytoscape and used to trace the seed genes. Because some seed genes are absent from the network, we could only consider the seed genes that are present. From a large number of genes to the final hub genes/biomarkers, the initial list of genes became smaller. The upregulated and downregulated networks were combined using Network Analyzer to form the merged network, shown in Figure 5.

**Figure 6:**
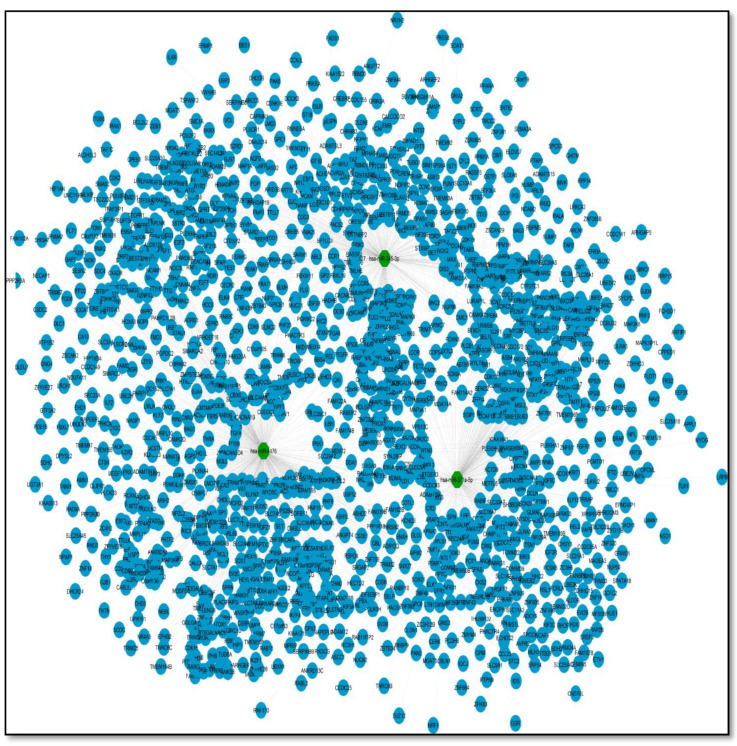
A miRNA–mRNA network of three key miRNAs from two CRS miRNA series. This represents a network of three miRNAs (selected on the basis of common miRNAs obtained from the two series). This network represents miRNAs with their respective targets. The total number of target genes of all three miRNAs are 1500 and the edges are 1609.

As a .sif file, Cytoscape created a miRNA–gene network utilizing the three miRNAs and their target genes in Table S4, Figure 6. Because miRNA molecules match the needed criteria of high sensitivity, specificity, and accessibility, they are regarded as suitable biomarkers. Many researchers have looked into the involvement of miRNAs in sepsis [47,48]. For example, Chen et al found that patients with sepsis had altered profiles of particular miRNAs and their targets [49]. Previous research has also found a link between the degree of miRNA expression and the mortality of sepsis patients [50,51].

**Figure 7:**
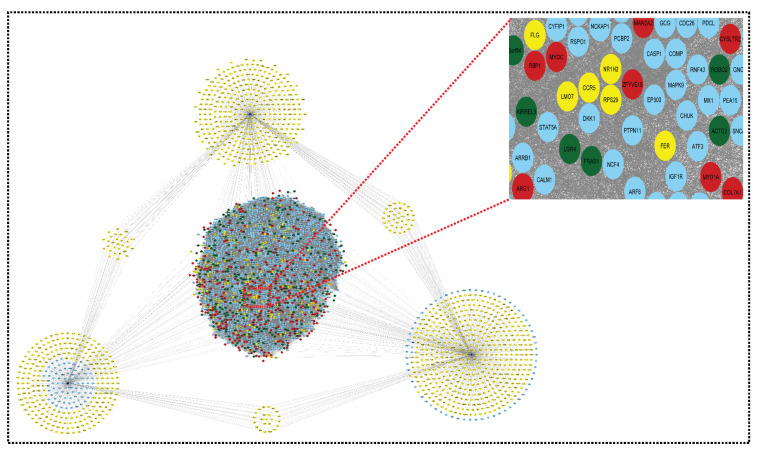
Shared network: this shared network is formed from all the above networks (upregulated network, downregulated network, and miRNA–mRNA network). It contains 2091 nodes and 75,957 edges. The red color indicates upregulated genes, the green color indicates downregulated genes, the yellow color indicates the target genes of miRNAs, and the blue color indicates the interacting genes.

In the merged network, this miRNA–mRNA network merges. Because the merged network already contains many target genes from the miRNA network, it is now known as a shared network. There are now 2091 nodes in the common network, with three miRNAs and 75757 edges (see Figure 7).

This complex network is then examined and used to obtain useful information about hub genes (based on centrality), seed genes tracking, gene enrichment analysis, and pathways. Overall, 45 modules are extracted from the native network. Out of the 45 modules, we chose the top ten and traced our seed genes. Finally, we used degree centrality to identify 27 seed genes from these 10 modules. We used degree centrality to extract the top 50 genes from each module while tracing our seed genes (see Table 3 Part A). 

**Figure 8:**
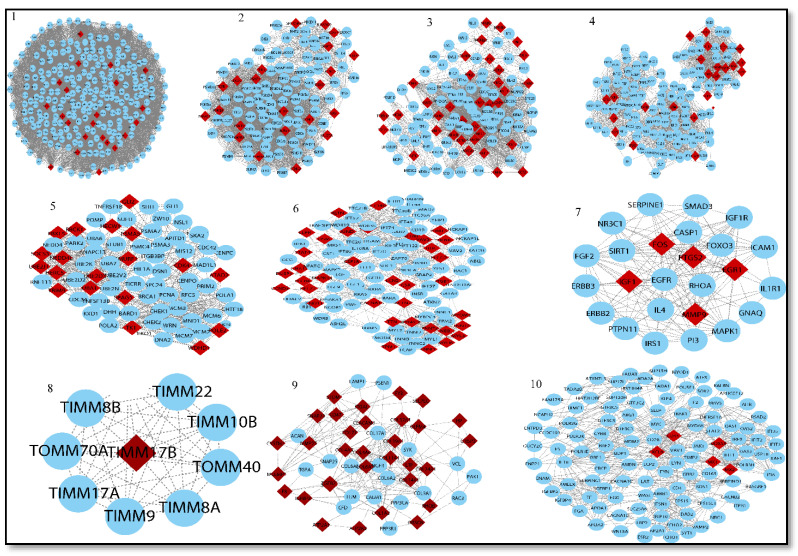
Top ten modules selected from MCODE analysis. Network/modules/sub-modules at different network levels that accommodate leading hubs (red) (fundamental key regulators) and blue nodes indicate interactive partners (other genes).

The final list (mixing the upregulated and downregulated genes) can be used to build the network. The total number of shared up- and downregulated genes in SLE and sepsis are 47 and 13, respectively, with 373 and 432 up- and downregulated genes in CRS Figure 8. 

**Figure 9:**
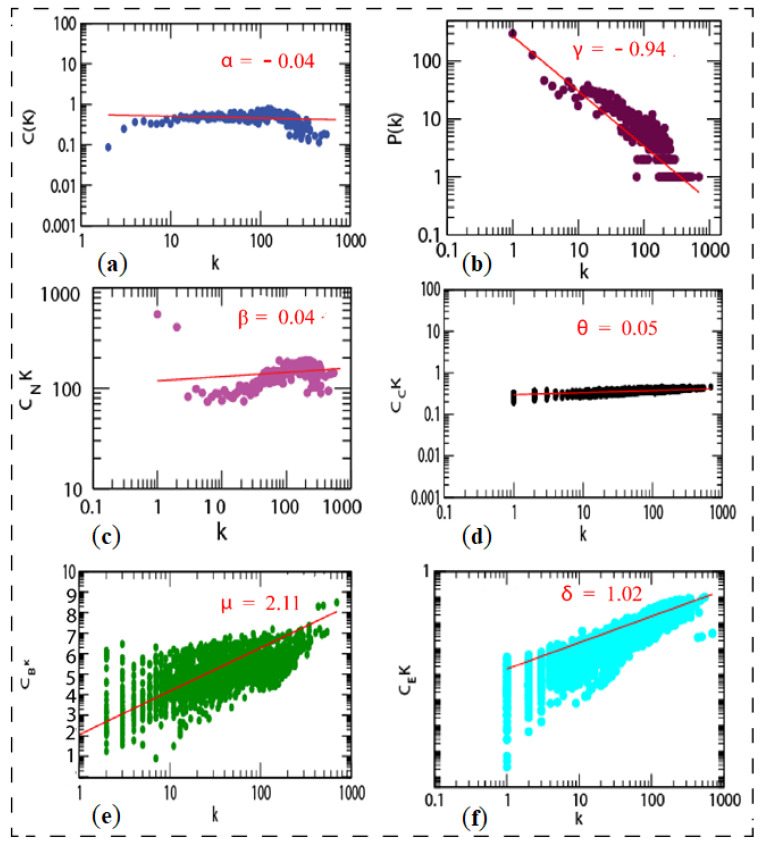
Topological properties of shared network: (**a**) clustering co-efficient (C(k)), (**b**) the behaviors of degree distributions (P(k)), (**c**) neighborhood connectivity (CN(k)), (**d**) closeness (CC(k)), (**e**) betweenness (CB(k)), and (**f**) eigenvector (CE(k)) measurements as a function of degree k for original shared network.

There was no need to integrate the network from STRING. However, we built the network separately for upregulated and downregulated genes, resulting in a higher number of non-seed nodes than in the mixed list. The more nodes there are, the more connections/interactions there are with the seed nodes. If there are a lot of nodes, it is possible that some of them are significant for the disease. If non-seed nodes are at the top of the list of hub nodes, there may be a biomarker that can be cross-checked in the literature to see what role that gene plays in the body. After identifying an interaction between them, we may rank the genes in the network using network centrality measures, such as degree, eigenvector, betweenness, and proximity centrality. Network representation and analysis are powerful tools for studying the complicated behavior of biological systems’ physiology and pathology. The CRS network was discovered to have a hierarchical scale-free nature through topological research. The network’s topological features show power law behavior, indicating that it is fractal in nature. The fractal form of the network’s self-affine process could be a symptom of the network’s self-organization (see Figure 9). 

These genes were utilized to build a gene network and to investigate their biological importance. We found the top 10 modules based on the MCODE analysis. Our genes of interest were found in each module. In these modules, we found 27 hub genes (see Table 3 Part B). We identified three miRNAs, namely, *hsa-miR-4476, hsa-miR-371a-5p*, and hsa-*miR-345-3p*. The miRNA–mRNA network was built using these miRNAs. Further, these networks were merged into a shared network to identify key fundamentals biomarkers. Based on degree centrality, we used each module and found out the hub genes. These are: *PSME3, CCNB1, CCNB2, BUB1, NCAPH, TTK., KIF11, NCAPG, FOS, EGR1, MMP9, IGF1, PTGS2, MX1, CXCL8, CD44, MX2, DDX58, COL1A2, COL11A1, COL5A2, COL6A3, COL4A6, COL4A5, ACTN2, TNNT3*, and *ATM*. Gene product interaction networks (PPI networks; G = (V, E)) are networks that are composed of gene products V and a collection of undirected interactions E, which are linked together by their connections to one another. The degree centrality of candidate genes is then ranked, and novel genes that are likely connected with the disease of interest are found based on the degree score. Based on degree centrality, 27 hub genes were selected, in which six genes (*MMP9, CD44, EGR1, ACTN2, TNNT3*, and *PTGS2*) were selected on the basis of a multi-network variable selection approach and validated in a well-established sepsis mice model. However, only three genes (*CD44, MMP9*, and *EGR1*) were found to be significantly upregulated in the sepsis model, correlating with our bioinformatic results. 

The most basic centrality measure is degree centrality, which is used to identify an important node participating in a large number of interactions. Because it is defined by the number of its neighbors, it is a local centrality measure. When it comes to the analysis of biological networks, it has been widely used. A higher degree centrality value indicates that a protein/gene is more likely to be required for the survival and growth of the organism, while a lower degree centrality value indicates that a protein/gene is not required. Since centrality measures are used to quantify the nodes or edges that are more important than others, degree centrality measures are utilized to numerically characterize the importance of genes in the biological system. 

These important regulators are firmly embedded in the system. They serve as the network’s backbone for all network activities and regulations, and they could be potential disease control target genes.

### 3.3. Gene Ontology of Top Hub Genes and Target Genes

**Figure 10:**
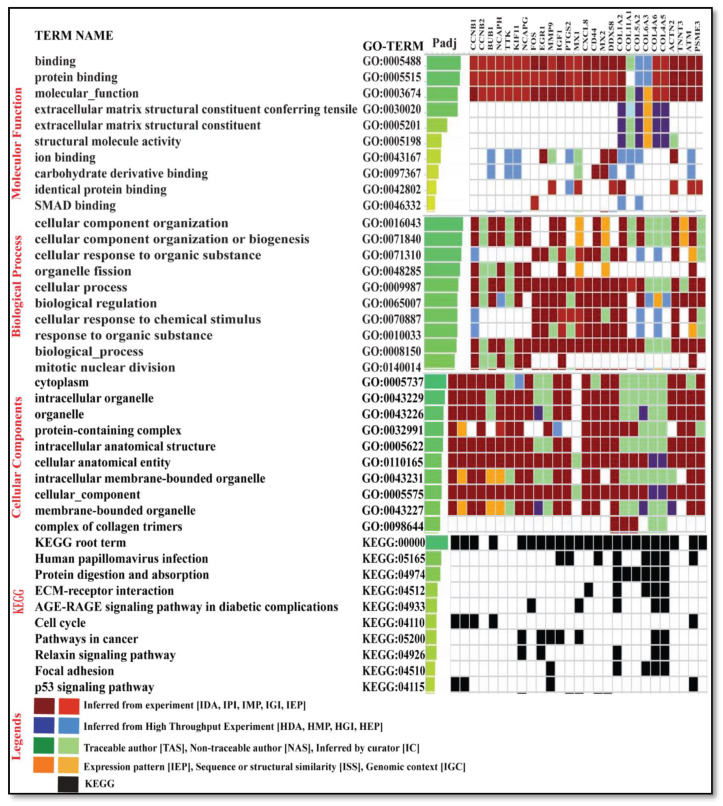
The figure depicts the top 10 gene ontology of key genes with GO term analysis ID and the padj molecular function, biological process, cellular components, and KEGG pathways.

A set of 27 genes were submitted to gProfiler for gene ontology term analysis (molecular function (MF), cellular component (CC), and biological process (BP)) and for pathway analysis using the enrichr database. Forming an idea of the effect of DEGs is only possible when we have some preliminary insight into their individual functions. The gene ontology (GO) term enrichment allows us to obtain information about the genes (see Table S5). The fundamental genes were found to display the differential expression irrespective of the condition, state of disease, and expression pattern (see Figure 10).

#### 3.4. qRT-PCR Validation 

**Figure 11:**
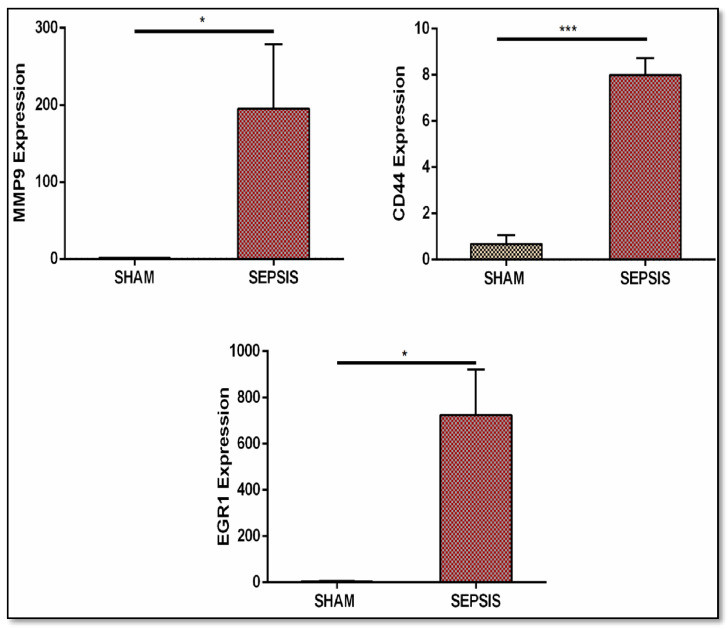
Validation through real-time qRT-PCR analysis of gene expression (*MMP9, CD44*, and *EGR1*). Sepsis groups are CLP operated and SHAM groups are control groups. Real-time analysis data showed their upregulated expression. Data are represented as mean ± S.E.M. * *p* < 0.05 and *** *p* < 0.001.

After an integrated bioinformatics approach, the key genes obtained were validated in a well-established sepsis mice model. qRT-PCR analysis was performed to quantify the relative fold change in expression.. Our findings through bioinformatics analysis and a validation study with qRT-PCR analysis in a well-established sepsis mice model were correlated (see Figure 11). 

## 4. Discussion

Sepsis is a life-threatening disorder caused by an aberrant immune response to infection that can result in tissue damage, organ failure, and death if not treated quickly and effectively [52]. When an infection spreads beyond local tissue containment, sepsis develops, resulting in a cascade of dysregulated physiologic responses and organ failure. Septic shock, which is marked by significant circulatory, cellular, and metabolic abnormalities, and which is associated with a higher mortality rate, affects a minority of patients with sepsis. The complicated interplay between the first inflammatory and later anti-inflammatory responses was blamed for sepsis-induced organ failure and death. Early 30-day sepsis mortality has decreased as a result of breakthroughs in intensive care medicine and goal-directed therapies, only to steadily increase long after "recovery" from acute events. Many researchers have focused on the long-term sepsis-induced abnormalities in cellular immune function, since so many sepsis survivors succumb to persistent, recurring, nosocomial, and secondary infections. Immune suppression, persistent inflammation, and bacterial persistence are all examples of how sepsis changes innate and adaptive immune responses over long periods of time following clinical recovery [53]. Over the past several decades, extensive basic and clinical research has elucidated underlying molecular pathways and inevitable mechanisms of sepsis. However, mortality and morbidity always remain high due to a lack of prompt treatment [54]. New technological explorations have identified various possible non-invasive treatments. In our study, the possible pathways to disseminate and control sepsis-related deaths were explored. Multifarious studies have demonstrated the intrinsic part miRNA plays in numerous sepsis-associated diseases, such as SLE [27,55] and CKD [56]. These altered miRNA levels can serve as diagnosis and prognostic markers of the diseases [57]. Significantly enriched levels of *miR-181a, miR-92a*, and *miR-424* in the blood of ARDS patients may serve as diagnostic markers, and were found to be closely associated with inflammation and epithelial cell injury [58]. In numerous sepsis-associated organ injuries, such as those to the lungs, kidneys, and other organs, miRNAs play an important role in cellular signaling, which then regulates the immune response and molecular pathological outcomes [59,60]. Additional studies revealed a significant decrease in the levels of miR-124 in the serum of SLE patients [61] and an upregulation of *miRNA-21* and *miR-214* in CRS and CKD patients, respectively [26,62]. 

Sixteen GSE series summarize the genomic expression profile of sepsis, SLE, and CRS. In the present study, a meta-analysis approach was adopted to identify the overlapping upregulated 47 DEGs and 13 overlapping downregulated DEGs in sepsis and SLE. All the DEGs (432 upregulated and 373 downregulated) of CRS and 252 target genes of three DEMs from the CRS series were taken. The STRING database was used to create separate upregulated and downregulated networks. Both the networks were then merged using a network analyzer tool, which we referred to as a merged network of genes. Only three overlapping DEMs, namely, *hsa-miR-345, hsa-miR-371a*, and *hsa-miR-4476*, from two GSE series were selected to create a miRNA–mRNA network that was further merged with the merged network of the genes, creating a very complex miRNA–mRNA interacting network, which we referred to as a shared network. Finally, 45 modules were extracted from the shared networks using MCODE, and the top 10 modules were selected based on the MCODE score and other centrality measures. Each module was further used for gene tracing (hub genes in each module) and a total of 27 hub genes were identified based on degree centrality (see Table 4).

The 27 hub genes (in which only six were genes from SLE and sepsis, while the other 21 were CRS DEGs) that we obtained were remarkably enriched in GO terms under biological processes. However, not so much research has been conducted on these, and as such there are few data related to CRS available that can be implemented to provide solid support for their interrelated manifestations in sepsis and SLE. Therefore, more research should be conducted to establish the interrelation. Moreover, we tried to establish one such interrelation in our study. Out of the six genes that overlapped in SLE and sepsis, three critical genes (*MMP9, CD44*, and *EGR1*) obtained through our bioinformatic approach were validated in a well-established sepsis mice model and were found to be upregulated. As sepsis, SLE, and CRS are interrelated, these genes may also be targeted in SLE and CRS.

In previous studies, *CRP, IL6*, and lactate could be used as biomarkers to evaluate the diagnosis and prognosis of sepsis severity in humans. Among them, IL6 is the most valuable biomarker for inflammation, and this has been reported in several studies [63]. In this study, the genes were identified when establishing a network for elucidating the common genes that might be involved in three inflammatory diseases (SLE, sepsis, and CRS). However, the severity of the disease requires periodical assessment. Here, we euthanized the mice after 16 hours following the CLP procedure and determined the expression of the genes, which limits our study.

Intruding bacteria and inhaled particles and chemicals first encounter alveolar macrophages, which provide the first line of defense. Macrophages recognize microorganisms non-specifically via pattern recognition receptors and initiate the secretion of inflammatory regulators in the lungs. Multiple pieces of evidence have confirmed that potassium channels are involved, and that lipopolysaccharides (LPS) induce the activation of macrophages. The ether-a-go–go-related gene (*ERG*) potassium channel belongs to the ether-a-go–go family of voltage-gated potassium channels. In preclinical models, targeted medicines aimed against EGFR, such as cetuximab and panitumumab, and those directed against VEGF, such as bevacizumab, have been demonstrated to generate a more immunogenic tumor profile, and so could be useful adjuncts to CRC [64]. The present study reinforces previously reported observations where *ERG1* has been overexpressed in different inflammatory diseases, including sepsis [65]. Therefore, *EGR1* can be a diagnostic marker for SLE and sepsis. Matrix metalloproteinases 9 (*MMP-9*) is a 92 kDa zinc-dependent gelatinase also known as type IV collagenase [66]. Secreted pro-*MMPs* converted to *MMPs* via enzymatic cleavage can influence the metastatic potency of immune cells by decreasing the expression of extracellular matrix proteins [66]. Earlier, *MMP-9* was reported to be a biomarker in severe sepsis patients, and can also serve as a biomarker for the severity of sepsis in pediatric patients [67,68]. A study by Alqahtani et al. found that the protein levels of *MMPs* was detected to be high in the lungs of an acute lung injury model induced by a cardiopulmonary bypass (CPB) [69]. 

The inhibition of MMPs reduces the lung inflammation mediated by neutrophils in a ventilator-induced lung injury model [70], thus increasing survival rates [71]. Moreover, *MMP9*-lacking septic mice had considerably decreased neutrophil infiltration, edema, and lung injury [72]. *IL-1β* is known as an inflammatory marker in various diseases. The overexpression of Il1b positively regulates *MMP9* expression and its catalytic activity. Moreover, *MMP9* cleaves IL-1β and regulates it negatively [73]. In abdominal sepsis, MMP-9 controls the shedding of platelet-derived CD40L and regulates neutrophil infiltration and recruitment to the lungs, which was found to be regulated by *MMP9* in abdominal sepsis model [72]. Lesiak A et al., observed a significantly higher level of *MMP9* in patients with systemic lupus erythematosus [74]. Etiologies such as sepsis were implicated in the activation of the RAGE-dependent NF-kB signaling cascade associated with inflammatory processes and oxidative stress [75]. Zhang H et al. reported that *MMP9* mediated the shedding of *RAGE*, and might exacerbate sepsis-associated pulmonary inflammation by controlling *RAGE/NF-κB* signaling [76]. *MMP9* is also considered to be a macrophage gene [77], where its overexpression in the lung tissue of septic mice model correlates with our study. Therefore, *MMP9* can serve as an effective bio-marker and prognosis assessment tool for inflammatory diseases. It is critical to recognize that some pharmacogenes, for which pharmacogenomic guidelines or prescription labels urge modifications in medical therapy in the context of specific genetic variants, are also known to impart greater disease risk. This is part of the study of how genes influence an individual’s response to pharmacological therapy (pharmacogenomics) [78]. CD44, a transmembrane protein receptor encoded by the *cd44* gene, synthesizes constitutively and presents several cell types, such as neurons, fibroblasts, leukocytes, keratinocytes, and epithelial cells. *CD44* is involved in cellular adhesion, migration, aggregation, and lymphocyte activation, such as with T cells and B cells. Various studies demonstrated *CD44*’s role in the extravasation and recruitment of lymphocytes to the lungs, kidneys, and joints in inflammatory diseases [79], including sepsis, chronic kidney disease [80], cardiac fibroblast [81,82], and SLE [83]. It binds to hyaluronic acid for downstream signaling, and is committed to host–pathogen interactions [84]. It has been reported that the inhibition of *CD44*’s function effectively reduced the buildup of neutrophils in the lungs in abdominal sepsis. Further study demonstrated that blocking *CD44* decreased pulmonary neutrophilia and also attenuated sepsis-induced lung edema and tissue injury [79]. It is also reported that an alteration in the distribution of *CD44* and hyaluronan has a consequential effect on SLE [83]. The inhibition of CD44 suppresses lymphoproliferation in lupus-prone mice. In a recent study, H. Fu et al. reported that targeting *CD44* can be a therapeutic approach in renal fibrosis and CKD [85]. In the present study, *CD44* was found to be significantly upregulated in the lungs of septic mice. However, further study and research are needed to establish the same trend in SLE and CRS models. Therefore, overexpressed *CD44* in SLE, sepsis, and CRS could serve as a disease biomarker. However, based on bioinformatics analysis, three unexplored miRNAs, *hsa-miR-4476, hsa- miR-371a-5p*, and *has-miR-345-3p*, were identified. However, it was previously reported that *miR-345-3p* reduces the inflammation caused by oxidized low-density lipoprotein. *miR-345-3p* plays a role in other diseases, such as diabetes mellitus [86,87], NSCLC lung carcinoma [88], heart failure [89], and glioblastoma [90]; its role in CRS, sepsis, and SLE, however, is unexplored. In their research, healthcare experts use a special category of literature applications, known as non-medical applications, which are extremely valuable for enhancing healthcare treatments and solutions to health problems [91]. Previously, a similar study was conducted by Fajarda et al., where nine datasets related to heart diseases were merged to obtain a gene expression signature (GES) set [92]. Such integrative analysis in the liver metastasis of colorectal cancer was performed [93]. Our study provides a general scheme for new approaches to uncover and understand the detailed and intricate structure of inflammatory diseases pathways. The datasets for sepsis and SLE are well balanced, whereas the dataset for CRS is very small and needs to be used with caution, even after the data have been normalized. For sepsis and SLE, we identified hub genes in the network, but there were no more (only three genes) common genes between the CRS GSE series. Therefore, we cannot compare the CRS common genes to sepsis and SLE. The number of genes in the datasets (16 GSE series) is taken into account in our research. Based on *p* values and fold-change criteria, we retrieved 3451 genes and 242 miRNAs, which we refer to as DEGs and DEMs, respectively, with 2276 upregulated and 1175 downregulated DEGs and 93 upregulated and 159 downregulated DEMs. To narrow down our research, we looked for overlapping genes in two diseases (sepsis and SLE), resulting in 60 overlapped DEGs. There were no overlapping genes in the two GSE series for CRS; therefore, we may use these CRS DEGs as references and integrate them with the 60 DEGs from the other GSE series. Only three genes (*EGR1, MMP8*, and *cd44*) were shown to be prevalent in the DEGs of sepsis, SLE, and CRS. We cannot build a PPIN network with only three genes. Therefore, we combined all of the CRS DEGs with the 60 DEGs (sepsis and SLE) and proceeded to create the PPI network using STRING. Furthermore, the top 10 modules were chosen from 45 modules and 27 hub genes, and three miRNAs from the shared network were discovered based on degree centrality. *EGR1* was the only gene that was found to be present in all three diseases, and it was also the gene that was extracted from the network based on degree centrality (which shows the highest impact of the node in the native network as a hub gene). This was further validated in the CLP-induced sepsis mice model where it was found to be significantly upregulated.

## 5. Conclusions

In summary, the present study aimed to identify and discover the potential biomarkers and associated pathways in sepsis, SLE, and CRS. An association between CRS type 5, sepsis, and SLE has been discovered in a number of studies. Because of these findings, one of our key objectives was to uncover genes that were shared by both sepsis and SLE. If these two diseases shared gene signatures with CRS, these genes would be used as critical genes in future studies. When the 60 genes revealed to be overlapping in sepsis and SLE were compared to the CRS DEGs, three important genes were discovered to be overlapping in CRS, SLE, and sepsis, according to our findings. Significant modules (strongly connected) were obtained from the networks (especially the miRNA–mRNA regulatory network). The key regulatory genes obtained through our multi-network variable selection approach were *MMP9, CD44*, and *EGR1*. These regulatory genes were validated in a CLP-induced sepsis mice model and were found to be significantly upregulated. As sepsis and CRS are interrelated, and because the occurrence of sepsis may cause/promote CRS, the results obtained emphasize the interrelated regulatory network of CRS, sepsis, and SLE, which may be used to suggest possible therapeutic targets for CRS in the future. The key regulatory genes reported in our study may help to unravel many unexplored regulatory pathways, leading to the identification of critical molecular targets for increased diagnosis, prognosis, and drug efficacy in CRS.

## Figures and Tables

**Table 1:** Details of GSE series sample and their DEGs and DEMs. Column 2 shows the total number of samples available in the particular GSE series, and column 7 indicates the fold change (FC) values. These GSE series were preprocessed using GEO2R, with many in-built functions for normalization and batch effect removal using empirical methods in limma. After applying the limma methods, the resultant data were obtained in the form of various calculated values, such as B value, *p*-Value, adj *p*-Value, fold change, and *t* values. The total up- and downregulated genes in each GSE series were written in columns 5 and 6, respectively. In this table, column 11 indicates the platform of the GSE series, which means that different platforms have different numbers of transcripts (Affymetrix Probe Set ID). GPL-570 always has 54675 transcripts, whereas GPL-4274 contains 18666 only.

SERIES	#Sample	Normal	Disease	Up	Down	FC	Illness	Country	Year	Platform	Author Reference
GSE6535	72	17	55	13	29	2	Sepsis	Australia	2007	GPL-4274	Tang BM [31]
GSE5772	94	23	71	28	19	0.5	Sepsis	Australia	2007	GPL-4274	Tang BM [31]
GSE28750	41	20	21	86	42	2	Sepsis	Australia	2011	GPL-570	Gareth price [32]
GSE64457	23	8	15	53	63	1.5	Sepsis	France	2015	GPL-570	Julien TT [33]
GSE12624	70	36	34	41	30	1	Sepsis	Germany	2010	GPL-4204	Hamid Hossain
GSE13205	21	8	13	107	81	0.5	Sepsis	UK	2008	GPL-570	Iain Gallagher [34]
GSE51997	36	22	14	662	205	0.5	SLE	Germany	2014	GPL-570	Powel Durek [35]
GSE13887	27	17	10	129	45	1.5	SLE	USA	2009	GPL-570	Frank A M [36]
GSE50772	81	20	61	169	53	1	SLE	USA	2015	GPL-570	Michael TS [37]
GSE30153	26	9	17	61	62	0.5	SLE	France	2003	GPL-570	Jean Nicolas [38]
GSE103760	16	8	8	489	103	1.5	SLE	USA	2018	GPL-17585	Morel L
GSE99967	59	17	42	65	11	0.5	SLE	Canada	2018	GPL-21970	Prokopec S [39]
GSE17582	48	12	36	11	10	1.5	CRS	Canada	2009	GPL-6883	Braam B [40]
GSE125898	6	3	3	362	422	2	CRS	Mexico	2019	GPL-10739	Rangel LA
GSE89699	8	1	7	0	81	0.5	CRS	India	2017	GPL-18402	Ramanathan k
GSE87885	5	3	2	93	78	0.5	CRS	China	2019	Gpl-22555	Rangjian Xu

**Table 2:** Real time pcr primers (mouse).

Genes	Forward	Reverse
*Cd44*	GAATTAGCTGGACACTCAAG	CACCTTCTCCTACTATTGACC
*Egr1*	CAGAGTCCTTTTCTGACATC	GAGAAGCGGCCAGTATAG
*MMP9*	CTTCCAGTACCAAGACAAAG	ACCTTGTTCACCTCATTTTG

**Table 3:** Part A: List of modules and their corresponding top 50 genes. Part B: Seed genes in top 10.

**PART A**		
**Modules**	**Count**	**Genes of Interest or Seed Genes (Top 50)**
module 1	22	*SRRM2, GSPT1, NOB1, SNRNP70, KRR1, SNRPA, SRSF1, POLR2K, CCT5, PPP2R1A, HNRNPC, UTP6, BYSL, RPL3L, RPL18, RPL38, EIF2S3, POLR2E, RPS27, SEC61A1, HEATR1, RIOK1*
module 2	22	*OASL, PSMD8, MPHOSPH6, PSME2, PSME3, CCNE2, BUB1B, UBE2D1, PLK1, ISG15, KCND3, CDKN1A, KIAA0020, CDC20, RAD23A, TCEB1, EXOSC5, PSME4, KCND2, RRP12, CCNA2, ANAPC4*
module 3	35	*ZWINT, KIF11, ASPM, CCNB1, RELA, KNTC1, NIFK, CENPN, TTK, BUB1, NFKB2, KIF20B, KIF2C, PRICKLE1, CKS2, NUF2, TP53, TYMS, ANLN, TDRD12, ECT2, NCAPG, CASC5, MDN1, TNF, ADRM1, CCNB2, KIAA0101, CDC7, CENPE, TTC27, NCAPH, CTNNB1, MCM10, NFKBIA*
module 4	24	*CX3CR1, CCL4L1, CCR3, RMI1, TAF1, EP300, ATM, TAF6, GNB2, TAF10, C3AR1, GNAI2, CCR2, GNG11, GTF2A1, ADCY1, CXCL1, CCL5, ATAD5, TAF13, PF4, GNG2, CXCL9, C3*
module 5	20	*PSMA5, TRIM9, GLI2, WDHD1, SOCS3, HERC5, CDK4, SMURF1, SPAG5, UBA1, HECW1, UBE2D3, ATAD2, TK1, FBXL5, EXO1, UBE2J1, RBCK1, NEDD4L, POLE2*
module 6	36	*JUND, IFT140, DUSP1, ITGB1, ACTN2, CSF1R, LEPR, CCND3, PRMT5, CACNA1S, IFT88, TNNT2, STAT4, PLCG2, NEB, VAV3, TPM1, TTN, TNNI1, PLCB4, MYOM2, MYBPC2, HELZ2, MYL3, LCN2, MYH6, BTK, CNOT1, MMP8, TNNT3, MAPK14, OSM, RETN, CSK, BLNK, GNAT2*
module 7	10	*FOS, IGF1, GNAQ, EGR1, PTGS2, IL1R1, NR3C1, PI3, MMP9, SMAD3*
module 8	49	*C4A, MX2, OAS2, IFI6, ENAM, VAMP2, OAS3, MX1, TF, FYN, ESR2, HIST2H2BE, IFI35, DAB2, OAS1, BDP1, XAF1, ARRB1, FCHO1, CYBB, SOS1, IFIT2, DDX58, IFIT1, RSAD2, NRG1, IFIT3, ENPP1, VAV1, LAT, LYN, FCHO2, ENTPD3, ARG1, APOA2, ITPR1, CXCL8, CD163, NCAPH2, ARHGEF12, RASGRF1, AMBN, CD44, USP18, TADA3, CD4, CACNA1C, GUCY2C, KALRN*
module 9	1	*TIMM17B*
module 10	33	*COL1A2, CD177, OLFM4, ITGAL, MCEMP1, ATP2A2, MMP25, TGFB2, SNAP23, CRISP3, COL12A1, IGFBP3, COL4A5, COL4A6, COL11A1, COL14A1, OBSCN, HP, FYB, ATP2A1, LTF, CEACAM8, CYSTM1, COL24A1, COL19A1, COL5A2, CD47, RHOB, F8, COL6A3, STOM, SPARC, SELPLG*
**PART B**		
**Modules**	**Count**	**Genes of Interest (Top 10)**
module 2	1	*PSME3*
module 3	7	*CCNB1, CCNB2, BUB1, NCAPH, TTK, KIF11, NCAPG*
module 4	1	*ATM*
module 6	2	*ACTN2, TNNT3*
module 7	5	*FOS, EGR1, MMP9, IGF1, PTGS2*
module 8	5	*MX1, CXCL8, CD44, MX2, DDX58*
module 10	6	*COL1A2, COL11A1, COL5A2, COL6A3, COL4A6, COL4A5*

**Table 4:** Twenty-seven hub genes were identified in the shared network based on degree centrality.

Sr. No.	Genes Symbol	Degree
1	*PSME3*	149
2	*CCNB1*	275
3	*CCNB2*	194
4	*BUB1*	242
5	*NCAPH*	132
6	*TTK*	151
7	*KIF11*	170
8	*NCAPG*	135
9	*ATM*	254
10	*ACTN2*	91
11	*TNNT3*	37
12	*FOS*	188
13	*EGR1*	126
14	*MMP9*	138
15	*IGF1*	120
16	*PTGS2*	116
17	*MX1*	57
18	*CXCL8*	246
19	*CD44*	134
20	*MX2*	39
21	*DDX58*	87
22	*COL1A2*	38
23	*COL11A1*	29
24	*COL5A2*	21
25	*COL6A3*	22
26	*COL4A6*	22
27	*COL4A5*	24

## Data Availability

The datasets used and/or analyzed during the current study are available from the corresponding author on reasonable request.

## Supplementary Materials

The following supporting information can be downloaded at: https://www.mdpi.com/article/10.3390/genes13020209/s1, Table S1: Total Genes of Sepsis in six GSE series, Table S2: Total Genes of SLE in six GSE series, Table S3: Overlapping Genes in Sepsis SLE and CRS, Table S4: Target Genes of key miRNAs using different database, Table S5: Gene enrichment term (GO-TERM) of key genes.
